# CBP501 suppresses macrophage induced cancer stem cell like features and metastases

**DOI:** 10.18632/oncotarget.19292

**Published:** 2017-07-17

**Authors:** Naoki Mine, Sayaka Yamamoto, Naoya Saito, Takuji Sato, Keiichi Sakakibara, Donald W. Kufe, Daniel D. VonHoff, Takumi Kawabe

**Affiliations:** ^1^ CanBas Co., Ltd., Numazu, Japan; ^2^ Dana-Farber Cancer Institute, Harvard Medical School, Boston, Massachusetts, USA; ^3^ Translational Genomics Research Institute (TGen), Phoenix, Arizona, USA

**Keywords:** macrophage, tumor microenvironment, cancer stem cell, paracrine, juxtacrine

## Abstract

CBP501 is an anti-cancer drug candidate which has been shown to increase cis-diamminedichloro-platinum (II) (CDDP) uptake into cancer cell through calmodulin (CaM) inhibition. However, the effects of CBP501 on the cells in the tumor microenvironment have not been addressed. Here, we investigated new aspects of the potential anti-tumor mechanism of action of CBP501 by examining its effects on the macrophages.

Macrophages contribute to cancer-related inflammation and sequential production of cytokines such as IL-6 and TNF-α which cause various biological processes that promote tumor initiation, growth and metastasis (1). These processes include the epithelial to mesenchymal transition (EMT) and cancer stem cell (CSC) formation, which are well-known, key events for metastasis.

The present work demonstrates that CBP501 suppresses lipopolysaccharide (LPS)-induced production of IL-6, IL-10 and TNF-α by macrophages. CBP501 also suppressed formation of the tumor spheroids by culturing with conditioned medium from the LPS-stimulated macrophage cell line RAW264.7. Moreover, CBP501 suppressed expression of ABCG2, a marker for CSCs, by inhibiting the interaction between cancer cells expressing VCAM-1 and macrophages expressing VLA-4. Consistently with these results, CBP501 *in vivo* suppressed metastases of a tumor cell line, 4T1, one which is insensitive to combination treatment of CBP501 and CDDP *in vitro*.

Taken together, these results offer potential new, unanticipated advantages of CBP501 treatment in anti-tumor therapy through a mechanism that entails the suppression of interactions between macrophages and cancer cells with suppression of sequential CSC-like cell formation in the tumor microenvironment.

## INTRODUCTION

Chronic inflammation can provide an environment conducive to tumor initiation and progression [1–4]. Tumor associated macrophages (TAMs) are a key promoter of cancer-related inflammation [5, 6]. Several reports have shown that a high density of TAMs is associated with poor prognosis and resistance to therapies [7, 8]. TAMs provide various cytokines and soluble factors that promote tumor growth and metastasis by promoting multiple biological processes, including cell proliferation, cell survival, angiogenesis, epithelial to mesenchymal transition (EMT) and cancer stem cell (CSC) formation [9]. EMT itself is often related to CSC state. CSCs are implicated in metastasis and resistance to multiple therapies [9, 10]. Based on these findings, TAMs are recognized as an important target for anti-tumor intervention [11, 12].

CBP501 is a peptide anti-cancer drug candidate, directly binds to Calmodulin (CaM) and inhibits CaM-mediated signaling [13]. This direct binding between CBP501 and CaM increases cis-diamminedichloro-platinum(II) (CDDP) uptake into cancer cells. However, the enhanced rate of CDDP uptake varies depending on the individual cell line [13, 14]. CBP501 has had two recently completed phase II clinical studies including one in Malignant Pleural Mesothelioma (MPM) and one in Non-small cell lung cancer (NSCLC). In phase I trials, the combination of CDDP plus CBP501 showed hints of clinical activity in patients with platinum-resistant ovarian carcinoma and MPM [15]. The primary endpoint, an increase in the rate of progression free survival at four months, was achieved in the Phase II study on MPM [16].

In this report, we investigated the role of CBP501 on the cells in the tumor microenvironment, independent of its effect on promoting CDDP uptake by cancer cells. Ex3ll Lewis, a lung carcinoma cell line, insensitive for CBP501-induced CDDP uptake, was employed in a co-culture experiment with the macrophage cell line RAW264.7. Additional *in vivo* experiments using mouse tumor models were also performed to elucidate the effects of CBP501 on the tumor microenvironment. CBP501 suppressed the production of cytokines by macrophages in a co-culture system. Furthermore, CBP501 suppressed juxtacrine interactions between Ex3ll cells expressing vascular cell adhesion molecule 1 (VCAM-1) and RAW264.7 cells expressing very late antigen-4 (VLA-4). Through these combined effects, CBP501 suppressed the induction of CSC-like features. Our results provide new insights into how CBP501 can affect the interaction between TAMs and cancer cells in the tumor microenvironment.

## RESULTS

### CBP501 suppresses production of cytokines and the expression of ABCG2 in a co-culture system of Ex3ll lewis lung carcinoma and the RAW264.7 macrophage cell line

**Figure 1:**
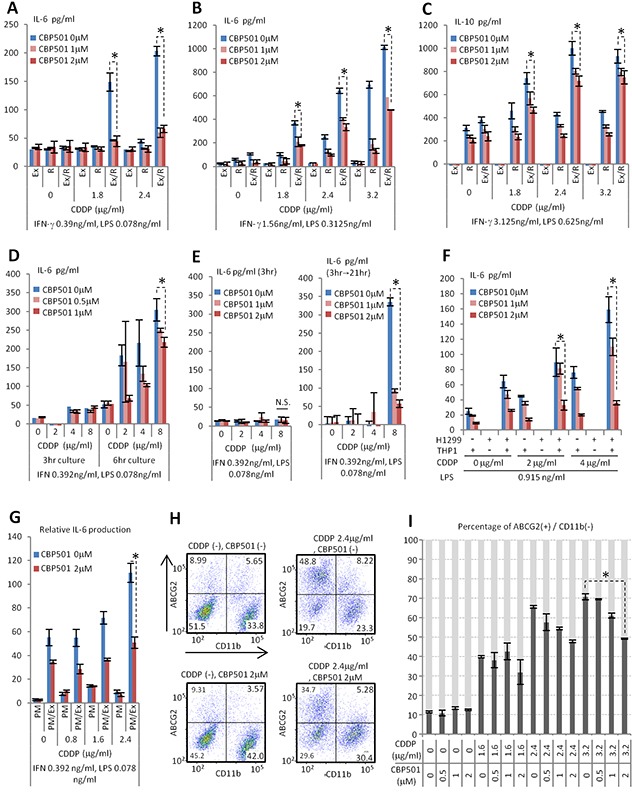
CBP501 suppresses production of cytokine and ABCG2 expression on cancer cell surface **(A-E)** ELISA assays for production of cytokines in co-culture system of Ex3ll Lewis lung carcinoma and RAW264.7 macrophage cell line (n=3). (A, B, D, E) IL-6. (C) IL-10. (A-C) 22 hr culture. **(F)** ELISA assay for production of IL-6 in co-culture system of NCI-H1299 NSCLC cell line and THP-1 monocyte/macrophage cell line (n=3). **(G)** ELISA assay for production of IL-6 in co-culture system of Ex3ll Lewis lung carcinoma and mouse primary peritoneal macrophage (n=3). (F, G) 22 hr culture. Ex, R, and PM mean Ex3ll, RAW264.7 and primary macrophage respectively. Ex/R and PM/Ex mean co-culture of Ex3ll and RAW264.7, and primary macrophage and Ex3ll, respectively. **(H, I)** Flow-cytometry analyses for ABCG2 and CD11b in co-culture of Ex3ll and RAW264.7 (48 hr culture) (n=3). (H) Dot-plots of flow-cytometry analyses. X axis is CD11b. Y axis is ABCG2. (I) Graph of flow-cytometry analyses. An asterisk indicates statistical significance (TTEST, p<0.01). N.S. = not significant (TTEST, p>0.05). Error bars indicate the standard deviation.

Macrophages comprise a substantial component of the tumor microenvironment [6]. To evaluate the drug effects of CBP501 on the tumor microenvironment, a co-culture system of the Ex3ll Lewis lung carcinoma with the RAW264.7 macrophage cell line was employed. Previous reports revealed that CBP501 increases the uptake of CDDP into cancer cells [13]. Ex3ll and RAW264.7 were examined to see whether they were sensitive to the combined CDDP/CBP501 treatment by examining CDDP-induced cytotoxicity with or without CBP501. Such cytotoxicity would be indicated by cell cycle subG1 and G2/M phase accumulation for Ex3ll or a WST assay for RAW264.7. These tests showed that both cell lines exhibited no difference between treatments with CDDP alone or the CDDP/CBP501 combination (Supplementary Figure 1). In related experiments, co-cultures of these cell lines in the presence of a low supplemental dose of Interferon-γ (IFN-γ) and LPS exhibited a CDDP dose-dependent increased production of Interleukin-6 (IL-6), Interleukin-10 (IL-10) and Tumor Necrosis Factor-α (TNF-α). In addition, Ex3ll was necessary for production of these cytokines. CBP501 suppressed production of these cytokines (Figure 1A-1C, Supplementary Figures 2 and 3). Earlier reports indicated that IL-6 and TNF-α have a tumor-promoting effect [17, 18]. IL-10 is known to lead to immune suppression [9]. Taken together, the results suggested that besides increasing CDDP-induced cytotoxicity, the anti-tumor effect of CBP501 might also arise in part by regulating the tumor microenvironment comprised minimally by cancer cells and macrophages. Next we investigated the time course for CDDP-induced IL-6 production in co-culture. The results indicated that increased IL-6 production occurs between three to six hours after CDDP treatment and that CBP501 suppresses this IL-6 production (Figure 1D and Supplementary Figure 2). Moreover, a 3 h combined treatment with CDDP/CBP501 had a lasting effect that persisted even at a time point observed 21 h after removing these drugs (Figure 1E and Supplementary Figure 2). We also demonstrated an effect of CDDP/CBP501 on IL-6 production in two other macrophage-cancer cell co-culture systems: (i) human NSCLC cell line NCI-H1299 with human macrophage cell line THP1 and (ii) Ex3ll with mouse peritoneal primary macrophages. The results were similar to that obtained with Ex3ll and RAW264.7 (Figure 1F, 1G), indicating that CBP501 suppresses the increased cytokine production by treatment with CDDP/IFN-γ/LPS.

Several reports have shown that IL-6 promotes the CSC-like state [19, 20]. The ATP-binding cassette sub-family G member 2 (ABCG2) is one of the markers for CSC and is related to drug resistance [21, 22]. We investigated ABCG2 expression in the cancer cell components of the co-culture systems. The myeloid lineage marker CD11b was used to separate Ex3ll and RAW264.7 cells by flow-cytometry (Figure 1H). Thus, the population that was simultaneously CD11b negative (−) and ABCG2 positive (+) is estimated to be “CSC-like”. Notably, CBP501 was found to suppress the formation of the CD11b (−)/ABCG2 (+) CSC-like population induced by CDDP/IFN-γ/LPS treatment (Figure 1H, 1I). Under the same conditions, however, a monoculture of Ex3ll showed an increase in ABCG2 (+) cells upon treatment with CDDP that was not suppressed by CBP501 treatment (Supplementary Figure 4). These results indicate that the CSC-like CD11b (−)/ABCG2 (+) population can arise by two mechanisms. The first is a cancer cell autonomous response to CDDP/IFN-γ/LPS. The second is dependent on the interaction between cancer cells and macrophages in response to CDDP/IFN-γ/LPS. CBP501 only suppressed CSC-like cells by the latter mechanism. Taken these together, CBP501 appears to suppress the formation of ABCG2 (+) CSC-like population that is induced by the interaction between Ex3ll and RAW264.7 cells in response to CDDP/IFN-γ/LPS.

### CBP501 suppresses cytokine production by targeting LPS-induced nuclear translocation of NFAT and NFκB in macrophages

**Figure 2:**
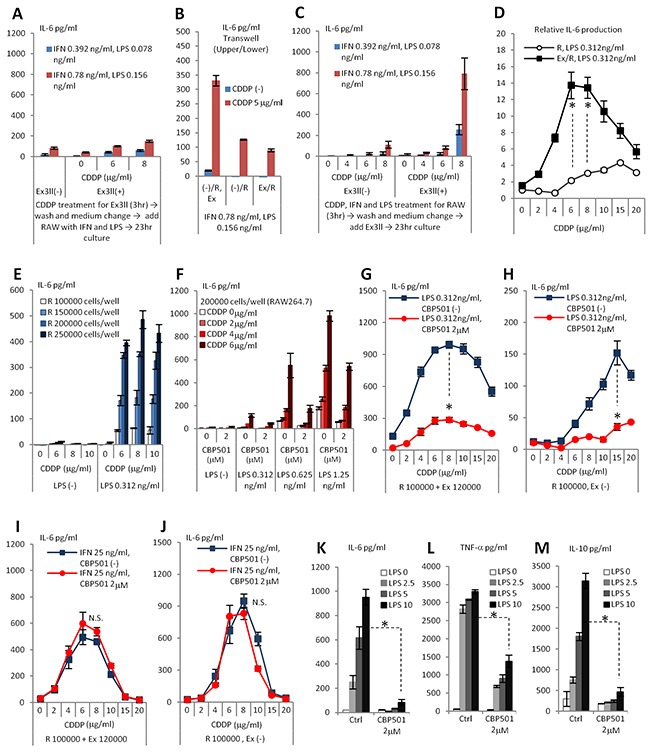
CDDP/IFN-γ/LPS treatment induces IL-6 production by affect to RAW264.7 rather than to Ex3ll And CBP501 suppresses LPS induced signaling in macrophage cell line. **(A-K)** ELISA assays for IL-6 production (n=3). **(L)** ELISA assay for TNF-α production (n=3). **(M)** ELISA assay for IL-10 production (n=3). (A) Only Ex3ll (120000 cells) was treated with CDDP for 3 hr. Then, medium was changed and RAW264.7 (100000 cells) was added with IFN-γ/LPS. (B) Only RAW264.7 (100000 cells) was treated with CDDP/IFN-γ/LPS for 3 hr. Then, medium was changed and Ex3ll (120000 cells) was added. (C) Trans-well experiment. (−)/R, Ex means no cells in upper-well and both RAW264.7 and Ex3ll are in lower-well. (−)/R means no cells in upper-well and only RAW264.7 is in lower-well. Ex/R means Ex3ll in upper-well and RAW264.7 is in lower-well. (D) Relative IL-6 production from RAW26.7 by CDDP/LPS treatment with or without Ex3ll in culture system. (E) Increased seeding cell number of RAW264.7 capacitates enough production of IL-6 by CDDP/LPS stimulation. (F) CBP501 suppresses CDDP/LPS induced IL-6 production in RAW264.7 single culture. (G, H) CBP501 does not affect to CDDP/IFN-γ induced IL-6 production. (G) Co-culture. (H) Only RAW264.7. (I, J) CBP501 suppresses CDDP/LPS induced IL-6 production. (I) Co-culture. (J) Only RAW264.7. (K-M) CBP501 suppresses LPS induced IL-6 production in RAW264.7 single culture. An asterisk indicates statistical significance (TTEST, p<0.0001). N.S. = not significant (TTEST, p>0.05). Error bars indicate the standard deviation.

In order to define the cellular target of CBP501 in the co-culture system, we focused on the mechanism of CDDP-induced cytokine production. Addition to RAW264.7 of conditioned medium from CDDP-treated Ex3ll did not induce IL-6 production by RAW264.7 (Data not shown). Additionally, the co-culture of RAW264.7 cells that had been pre-treated with a low dose IFN-γ/LPS and Ex3ll cells that had been pre-treated with CDDP for 3 h also did not induce IL-6 production (Figure 2A and Supplementary Figure 2). Moreover, a transwell experiment, which kept the different cell types separated but in a shared medium, showed that the presence of Ex3ll in the upper well did not enhance the CDDP/LPS-induced production of IL-6 by RAW264.7 in the lower well (Figure 2B). On the other hand, addition of Ex3ll cells directly to RAW264.7 cells that had been pre-treated for 3 h with CDDP plus low dose IFN-γ/LPS did indeed induce IL-6 production (Figure 2C and Supplementary Figure 2). These results suggest that the induced production of cytokines by CDDP/IFN-γ/LPS in the co-culture system occurs mainly through CDDP/IFN-γ/LPS's effects on macrophages rather than on cancer cells. However, the results demonstrate that the cancer cells do have a priming role for the macrophage's response to CDDP/LPS (Figure 2D), an effect that requires direct contact (Figure 2C). Next we evaluated the roles of IFN-γ and LPS in IL-6 production in the co-culture system. Dose escalation with LPS alone, but not with IFN-γ alone, induced robust production of IL-6 in co-culture at a basal level of CDDP (2μg/ml) (Supplementary Figure 5). The addition of IFN-γ was, in fact, found to be unnecessary for CDDP-induced IL-6 production in co-culture (Figure 2D, 2E). Although cancer cells appear to be required for the response of macrophages to CDDP/LPS, we tried to exclude the cancer cells from co-culture system to see if we could further simplify the system. In fact, increasing the seeding number of RAW264.7 cells was able to increase the level of IL-6 induced by CDDP/LPS (Figure 2E) and CBP501 was able to suppress this IL-6 production by cells grown at increased seeding number (Figure 2F). These results indicate that CBP501 suppresses IL-6 production by targeting macrophages that have been stimulated with CDDP/LPS. We then evaluated the effect of added CBP501 on the combination of LPS (0.312ng/ml) and CDDP in the presence or absence of Ex3ll cells. The results showed robust suppression of CDDP/LPS-induced IL-6 production in either case (Figure 2G, 2H). On the other hand IL-6 production induced by CDDP/IFN-γ co-treatment, could not be robustly suppressed by CBP501 (Figure 2I, 2J). These results suggest that CBP501 mainly targets LPS induced signaling rather than that induced by CDDP and IFN-γ. In fact, even in the absence of CDDP, CBP501 suppressed production of cytokines induced by high dose LPS stimulation alone in RAW264.7 monoculture (200000 cells/well) (Figure 2K-2M).

**Figure 3:**
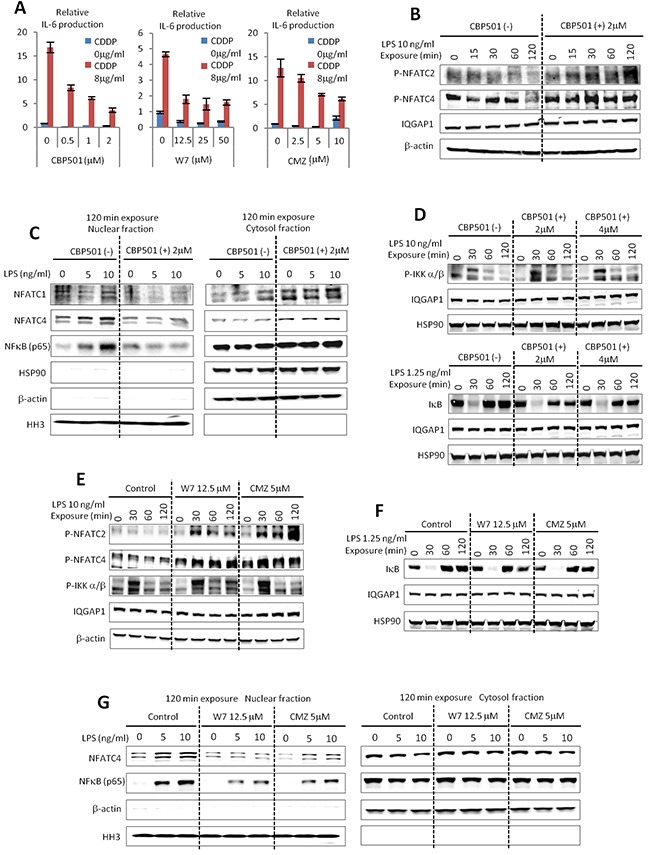
CBP501 suppresses nuclear translocation of NFATs and NFκB through CaM inhibition **(A)** CaM inhibitors of W7 and Calmidazolium (CMZ) suppressed IL-6 production by CDDP/IFN-γ (0.78 ng/ml)/LPS (0.15 ng/ml) stimulation similar to CBP501 (n=3). **(B)** Western blot analyses for phospho-NFATC2 and phospho-NFATC4 in RAW264.7 cells which were treated with LPS/CBP501 and collected at indicated time point. **(C)** Western blots analyses for NFATC1, NFATC4 and NFκB in nuclear fraction and cytosol fraction of RAW264.7 cells. RAW264.7 cells were fractionated at 120 min after treatment of LPS/CBP501. **(D)** Western blot analyses for phospho-IKK α/β and IκB in RAW264.7 cells which were treated with LPS/CBP501 and collected at indicated time points. **(E, F)** Western blot analyses for phospho-NFATC2, phospho-NFATC4, phospho-IKK α/β and IκB in RAW264.7 cells which were treated with LPS/W7 or CMZ and collected at indicated time points. **(G)** Western blots analyses for NFATC4 and NFκB in nuclear fraction and cytosol fraction of RAW264.7 cells. RAW264.7 cells were fractionated at 120 min after treatment of LPS/W7 or CMZ. (B, D, E, F) IGGAP1, β-actin and HSP90 were used as internal control to see loading protein amounts. (C, G) Histone H3 (HH3) was used as internal control for nuclear fractions. Heat-shock protein90 (HSP90) and β-actin were used as internal control for cytosol fractions.

In previous reports, we established that CBP501 binds to CaM and showed some similarities to other CaM inhibitors [13]. It was of interest then to see whether other CaM inhibitors W7 and Calmidazolium (CMZ) exhibited similar effects on IL-6 production by macrophages. Indeed, these other CaM inhibitors showed suppression of IL-6 production similar to CBP501 in the co-culture system (Figure 3A). These results indicate that CaM inhibition by CBP501 treatment is involved in suppressing IL-6 production. The protein nuclear factor of activated T cells (NFAT) is known to be a CaM/Calcineurin regulated transcription factor. De-phosphorylation of NFAT by CaM/Calcineurin promotes NFAT nuclear translocation [23]. Several reports indicated that activation of NFAT is involved in the cytokine production from macrophages [24–26]. To assess the role that NFATs may play in the induction and suppression of IL-6 in macrophages, we examined changes phospho-NFAT levels by western blot analysis under several different treatment conditions. It was found that the presence of CBP501 allowed RAW264.7 cells to retain high levels of phospho-NFATs upon LPS treatment (Figure 3B). Next we examined the nuclear versus cytoplasmic localization of NFATs in LPS-treated RAW264.7 cells. The results indicated that CBP501 treatment suppresses nuclear translocation of NFATs (Figure 3C). Interestingly, nuclear translocation of nuclear factor-kappa B (NFκB) was also suppressed by CBP501 treatment (Figure 3C). CaM has in fact been previously implicated in the activation of NFκB signaling [27, 28] and NFκB is one of the major transcription factors that mediates LPS-activated Toll like receptor (TLR) signaling [29]. Nuclear translocation of NFκB is usually negatively regulated by binding to Inhibitor of kappa B (IκB). Activation of TLR4 by its ligands such as LPS phosphorylates IκB kinase (IKK) to activate IKK. Phosphorylation of IkB leads to IκB degradation, which in turn causes nuclear translocation of NFκB [30]. Nuclear NFκB induces the expression of cytokines and IκB [29–31]. IκB induction by NFκB functions as a negative feedback system to excessive IKK-NFκB signaling [31]. In the experiments here, CBP501 treatment did not suppress LPS-induced IKK phosphorylation (Figure 3D); IκB degradation occurred within thirty minutes after LPS treatment and was not affected by CBP501 treatment (Figure 3D). However, feedback recovery of IκB protein levels, which normally occurs starting sixty minutes after LPS treatment, was reduced by CBP501 treatment (Figure 3D). These results suggest that CBP501 treatment inhibits nuclear translocation of NFκB without affecting upstream signaling. Moreover, CaM inhibitors W7 and CMZ had effects similar to those of CBP501 on IKK phosphorylation, IκB protein levels and nuclear translocation of NFκB (Figure 3E-3G). These results indicate that CaM inhibition by CBP501 leads to suppressed nuclear translocation of NFκB.

### CBP501 suppresses cancer spheroid formation that is induced by a macrophage mediated paracrine mechanism

**Figure 4:**
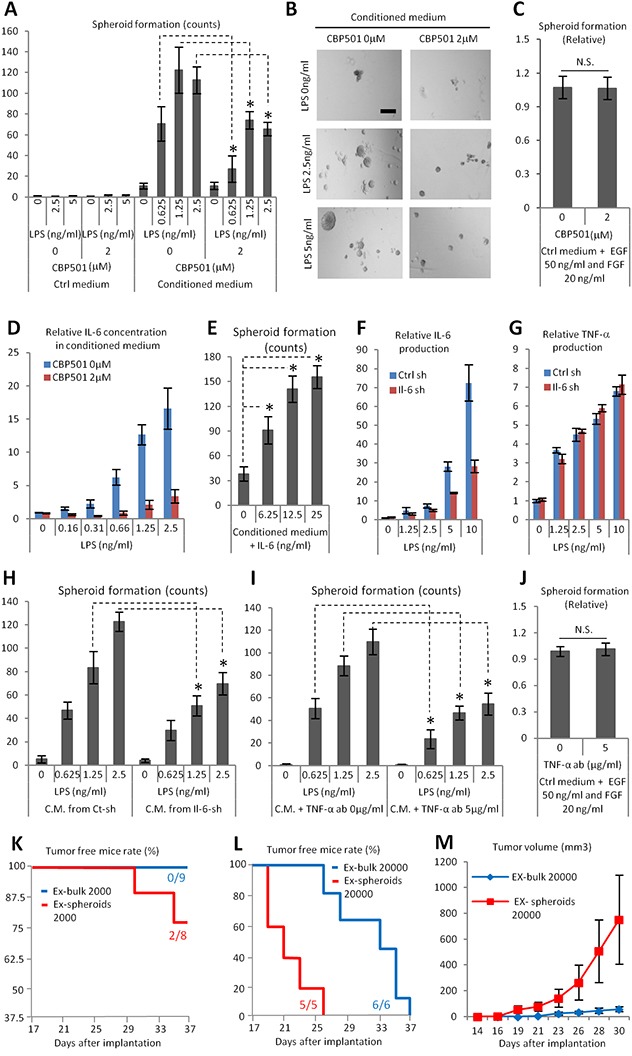
CBP501 suppresses cancer spheroid formation which induced by paracrine effect by macrophage cell line RAW264.7 (A-C, E, H, I, J) Ex3ll spheroid formation assay **(A)** Graph of Ex3ll spheroid formation in conditioned medium from LPS/CBP501 treated RAW264.7 (n=6). (B) Representative images of spheroid formation from A. Scale bar indicates 200μm. (C) CBP501 did not affect to spheroid formation induced by EGF/FGF (n=8). (D) ELISA assay for IL-6. Confirmation of production of IL-6 in the conditioned medium which were applied for A. (E) Graph of Ex3ll spheroid formation in conditioned medium with mouse recombinant IL-6. **(F)** ELISA assay for IL-6. Production of IL-6 in both control-sh (Ctrl-sh) and IL-6-sh (Il-6-sh). **(G)** ELISA assay for TNF-α. Production of TNF-α in both control-sh (Ctrl-sh) and IL-6-sh (Il-6-sh). (H) Graph of Ex3ll spheroid formation in conditioned medium from Ctrl-sh and Il-6-sh (n=6). (I) Graph of Ex3ll spheroid formation in conditioned medium with or without TNF-α neutralizing antibody (TNF-α-ab) (n=6). (J) TNF-α-ab did not affect to spheroid formation induced by EGF/FGF (n=8). (K-M) Bulk Ex3ll or sheroid formed Ex3ll were subcutaneously implanted to frank of SCID mice. **(K, L)** Tumor (50 mm^3^ <) free survival curves. (K) 2000 cells (Log-rank test, p=0.095). (L) 20000 cells (Log-rank test, p=0.0017). **(M)** Formed tumor growth from L. An asterisk indicates statistical significance (TTEST, p<0.0005). N.S. = not significant (TTEST, p>0.05). Error bars indicate the standard deviation for A-J. Error bars indicate the standard error for M.

We reasoned that CBP501 might suppress the formation of ABCG2 (+) CSC-like cells that are induced by the interaction between Ex3ll and RAW264.7 cells. There are three important features of CSC-like cells: 1) anchorage independent cell proliferation; 2), tumor initiation; and 3) drug resistance [32–34]. We assessed CBP501′s effect on anchorage independent cell proliferation by using conditioned medium from LPS treated RAW264.7 cells with or without CBP501. Spheroid formation was increased in conditioned medium collected from LPS-stimulated RAW264.7 and CBP501 suppressed this conditioned medium-dependent spheroid formation (Figure 4A, 4B). CBP501′s effect was also assessed under another condition [35] leading to spheroid formation; that is the addition of EGF and FGF. CBP501 did not affect spheroid formation under this second set of conditions (Figure 4C). These results lead us to hypothesize that CBP501 suppresses cancer spheroid formation indirectly by suppressing effects induced by soluble factors from RAW264.7 cells. IL-6 is one factor for which production in conditioned medium was suppressed by CBP501 (Figure 4D). Furthermore, addition of recombinant IL-6 to conditioned medium from RAW264.7 cells grown without added LPS still exhibited increased spheroid formation (Figure 4E). In order to demonstrate the role of IL-6 in conditioned medium from LPS-treated RAW264.7 cells, we constructed RAW264.7 cells with IL-6 knocked down (IL-6-sh) by means of lentivirus particles. About 50% knock-down of IL-6 and no change of TNF-α production was confirmed (Figure 4F, 4G). Relative to conditioned medium from the control RAW264.7 cells, spheroid formation in conditioned medium from IL-6-sh was reduced (Figure 4H). Moreover, the requirement of TNF-α for spheroid formation was assessed by using a TNF-α neutralizing antibody. Addition of TNF-α neutralizing antibody into conditioned medium from LPS treated RAW264.7 cells reduced the formation of spheroids (Figure 4I), but TNF-α neutralizing antibody did not show any effect on spheroid formation induced by co-addition of EGF and FGF (Figure 4J). These results suggest that IL-6 and TNF-α from macrophages are external factors required for cancer spheroid formation. To assess the tumor initiation competency of the formed spheroids, bulk cancer cells or cell spheroids were implanted by subcutaneous injection into the flanks of SCID mice. Cells from third generation spheroids, which were cultured in conditioned medium from the macrophage cell line, showed increased tumor initiation competency compared to bulk cancer cells (Figure 4K-4M). Thus, cancer cells can acquire at least the anchorage-independent proliferation and tumor initiation properties of CSC-like cells by interacting with macrophages through a paracrine mechanism. CBP501 can suppress the acquisition of these CSC-like properties by suppressing the production of cytokines by macrophages, at least the production of cytokines IL-6 and TNF-α as demonstrated here.

### CBP501 suppresses ABCG2 expression in cancer cells through the suppression of attachment dependent mechanism between cancer cell and macrophage

**Figure 5:**
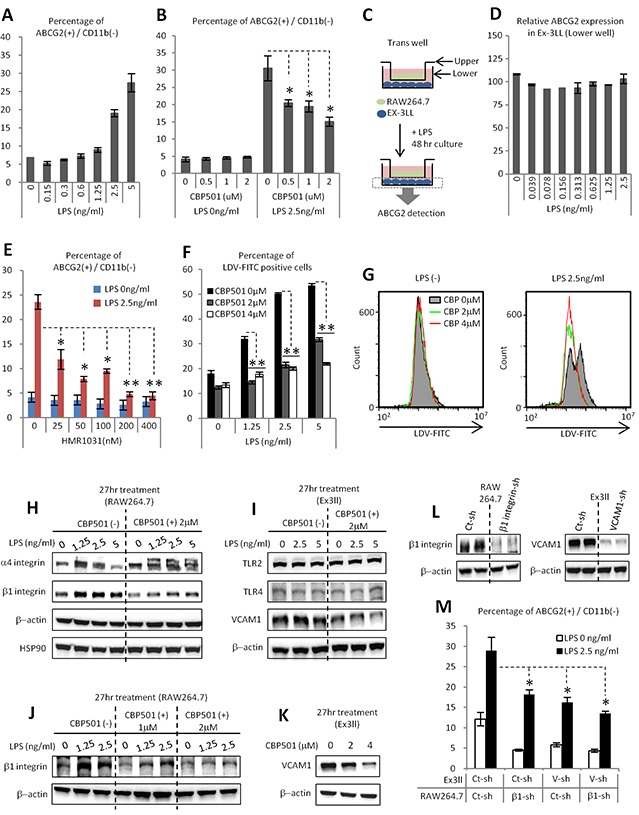
CBP501 suppresses ABCG2 expression in cancer cell through the suppression of attachment dependent mechanism between cancer cell and macrophage **(A, B, D-F, M)** Flow-cytometry analyses (n=3).(A) LPS stimulation increases ABCG2 expression on cancer cell surface in co-culture system of Ex3ll and RAW264.7 (48 hr culture). (B) CBP501 suppresses LPS induced ABCG2 expression on cancer cell surface in co-culture system. **(C)** Experimental scheme of D (trans-well culture). (D) LPS does not increase ABCG2 expression on cancer cell surface in trans-well system. (E) HMR1031 suppresses LPS induced ABCG2 expression on cancer cell surface in co-culture system. (F) CBP501 suppresses LPS induced LDV-FITC binding to RAW264.7 (24 hr culture). **(G)** Histograms of flow-cytometry analyses in F. **(H-L)** Western blot analyses. HSP90 and β-actin were used as internal control. (M) Knock-down of b1-integrin in RAW264.7 and VCAM-1 in Ex3ll suppresses ABCG2 expression. An asterisk indicates statistical significance (TTEST, p<0.0005). Two asterisks (TTEST, p<0.00005). N.S. = not significant (TTEST, p>0.05). Error bars indicate the standard deviation.

Although we demonstrated that cytokine production by macrophages induces CSC-like properties, such as anchorage independent proliferation and tumor initiation in cancer cells, the mechanism of induction of the drug resistance marker ABCG2 remained unexplained. LPS stimulation alone could induce ABCG2 expression in Ex3ll cells in the co-culture system (Figure 5A). CBP501 treatment could suppress this expression of ABCG2 (Figure 5B). We first hypothesized that induction of ABCG2 might also be induced by a paracrine effector from macrophages. To examine this hypothesis, Ex3ll cells were cultured in conditioned medium collected from LPS-treated RAW264.7. However, expression of ABCG2 in Ex3ll cells grown in this medium was unaltered (Supplementary Figure 6). Moreover, co-cultures using physically separated macrophages in the transwell system also exhibited no change of ABCG2 expression in the Ex3ll cells (Figure 5C, 5D). These results suggest that ABCG2 expression in cancer cells in the co-culture system requires direct cell-cell contact between cancer cells and the macrophages, rather than an indirect cytokine-mediated paracrine mechanism. We therefore searched for possible juxtacrine mechanisms by which ABCG2 expression might be induced. One earlier report indicates that the interaction between VCAM-1 and CD44 on a cancer cell's surface can induce ABCG2 expression [36]. VCAM-1 is a known mediator of cell adhesion through its binding to VLA-4 (α4/β1-integrin) [37]. β1-integrin (VLA-4, β-subunit) can be induced by NFκB signaling [38]. We hypothesized that interaction between cancer cells expressing VCAM-1 and macrophages expressing VLA-4 might induce expression of ABCG2 in the cancer cells. HMR1031 is a specific inhibitor of the interaction between VCAM-1 and VLA-4 [37]. HMR1031 was therefore added to the co-culture system to examine the hypothesis. Notably, HMR1031 strongly suppressed LPS-induced ABCG2 expression in the cancer cell population (Figure 5E). In order to investigate the effect of CBP501 on the interaction between cancer cells expressing VCAM-1 and macrophages expressing VLA-4, LDV-FITC was used to detect the expression of VLA-4 protein on the macrophage cell surface. LDV-FITC is a fluorescent ligand specific for VLA-4 [39]. Increased binding of LDV-FITC to RAW264.7 cells was observed after LPS treatment of these cells. In contrast, CBP501 suppressed this binding of LDV-FITC to RAW264.7 (Figure 5F, 5G). CBP501 treatment of RAW264.7 cells also suppressed LPS-induced β1-integrin expression (Figure 5H, 5J). Moreover, CBP501 treatment suppressed VCAM-1 expression in Ex3ll cells (Figure 5I, 5K). Furthermore, knock-down of either β1-integrin in RAW264.7 cells or of VCAM-1 in Ex3ll cells suppressed ABCG2 expression in Ex3ll cells in the co-culture system (Figure 5L, 5M). Taken together, these results show that CBP501 can suppress ABCG2 expression in cancer cells by suppressing cell-cell interactions between cancer cells expressing VCAM-1 and macrophages expressing VLA-4, at least by reducing the expression levels of these surface molecules.

### CBP501 suppresses formation of ABCG2 positive cancer cell population in a xenograft model

**Figure 6:**
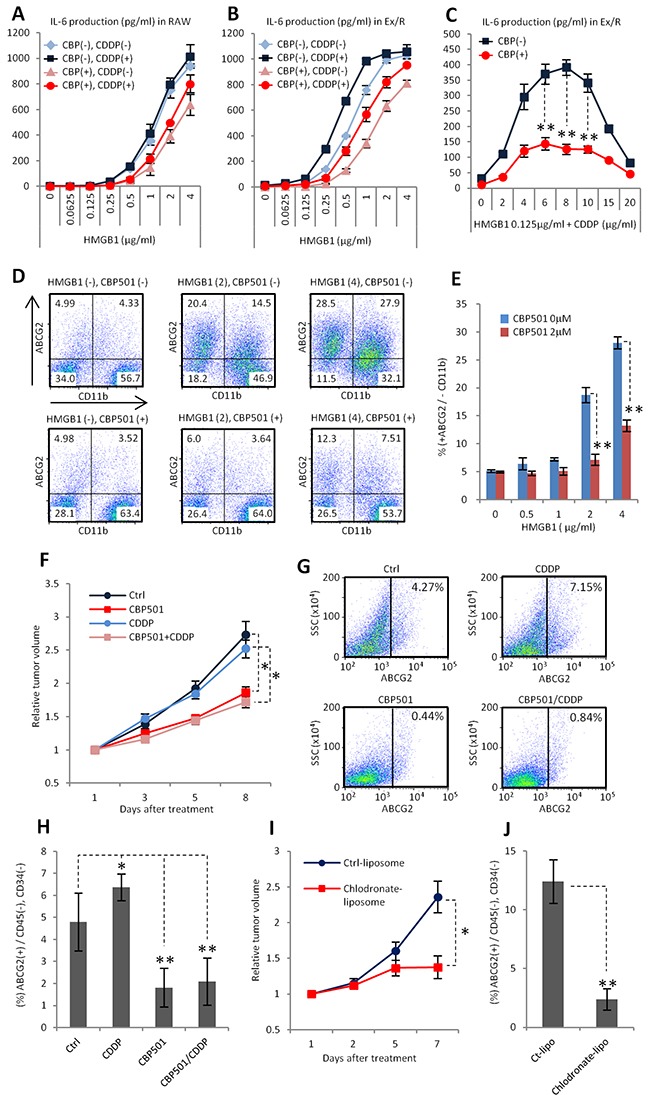
CBP501 suppresses formation of ABCG2 positive cancer cell population in xenograft model **(A-C)** ELISA assays for IL-6 production (n=3). CBP (−) and (+) mean CBP501 0μM and 2 μM respectively. CDDP (−) and (+) means CDDP 0mg/ml and 2 mg/ml respectively. **(D, E)** Flow-cytometry analyses for ABCG2 and CD11b in co-culture of Ex3ll and RAW264.7 (48 hr culture) (n=3). (D) Dot-plots of flow-cytometry analyses. X axis is CD11b. Y axis is ABCG2. (E) Graph of flow-cytometry analyses of D. **(F)** Graph of relative tumor volume change (more than twelve tumors in each treatment groups). Ex3ll cells were implanted into the flank of C57BL/6 mouse by subcutaneous injection. 7.5mg/kg of CBP501 was administrated by i.v. injection everyday (first to fifth day). 3mg/kg of CDDP was administrated by i.p. injection at fifth day. **(G, H)** Flow-cytometry analyses for ABCG2 expression on cancer cell surface in grafted tumors. Grafted tumors were dissected at eighth day from starting day of treatment. (G) Dot-plots of flow-cytometry analyses. X axis is ABCG2. Y axis is side scatter (SSC). (H) Graph of flow-cytometry analyses of G. **(I)** Graph of relative tumor volume change (six tumors in each treatment groups). 200μg/mouse of control-liposome or chlodoronate-liposome was administrated by i.v. injection at first and fifth day. **(J)** Flow-cytometry analyses for ABCG2 expression on cancer cell surface in grafted tumors of I. Grafted tumors were dissected at seventh day from starting day of treatment. An asterisk indicates statistical significance (TTEST, p<0.005). Two asterisks (TTEST, p<0.0005). N.S. = not significant (TTEST, p>0.05). Error bars indicate the standard deviation for A-C, E, H and J. Error bars indicate the standard error for F and I.

We demonstrated that the interaction between cancer cells and macrophages promotes formation of CSC like properties in an *in vitro* co-culture system. To evaluate the role of this mechanism in an actual tumor, HMGB1, which is one of the endogenous ligands for TLR2/4, was added to the co-culture system instead of LPS. In tumors, the main source of HMGB1 is through release from necrotic cells, which are present under conditions of low nutrient and oxygen levels [40]. Moreover, macrophages favor such conditions [41]. In our experiments, similarly to LPS, HMGB1 induced IL-6 production synergistically with CDDP in both isolated RAW264.7 cells and in the co-culture system. CBP501 treatment suppressed this IL-6 production (Figure 6A-6C). Moreover, the expression of ABCG2 was induced by HMGB1 and CBP501 treatment suppressed this enhanced expression (Figure 6D, 6E). These results suggest that HMGB1 is one of the important stimuli for macrophages to promote the formation of CSC-like cells in actual tumors. Next, we investigated the effect of CBP501 on ABCG2 expression *in vivo* in an Ex3ll tumor xenograft. Sequential administration of CBP501 for five days retarded tumor growth, but a low dose (3 mg/kg) of CDDP had no effect (Figure 6F). In these experiments, CDDP administration slightly increased ABCG2 expression in CD45(−) and CD34(−) (immune-cell and endothelial markers described in ref. 42) cancer cell sub-populations (Figure 6G, 6H). In contrast, administration of CBP501 suppressed ABCG2 expression in the cancer cells (Figure 6G, 6H). Elimination of TAMs by dosing with chlodronate-liposome [43] was performed to evaluate the role of TAMs for ABCG2 expression in cancer cells. Elimination of TAMs was found to retard tumor growth and to suppress ABCG2 expression in cancer cells, similar to the effects of CBP501 (Figure 6I, 6J). These results suggest that CBP501 suppresses formation of ABCG2 positive CSC-like cells in the grafted tumor by suppressing interactions between cancer cells and TAMs.

### CBP501 suppresses metastasis in lung metastasis model by grafting 4T1 breast cancer cell line

**Figure 7:**
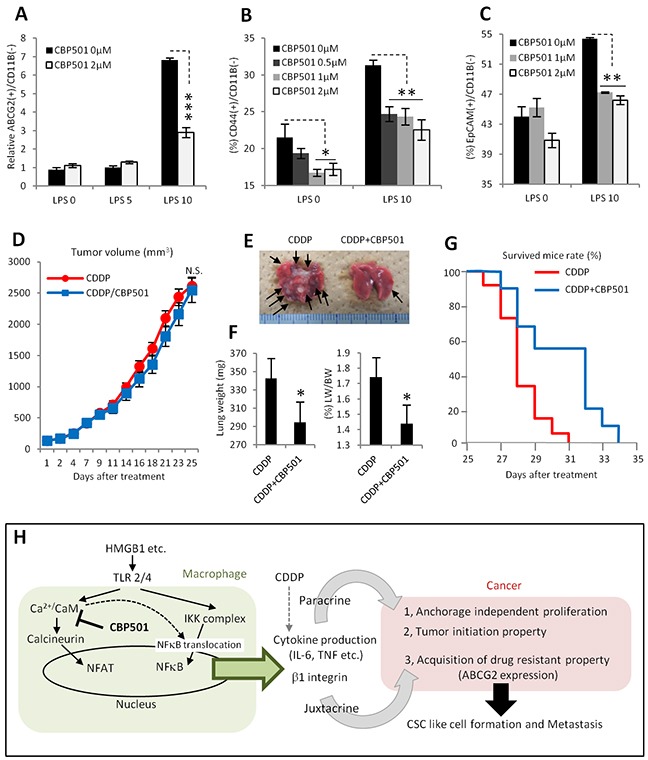
CBP501 suppresses metastasis in lung metastasis model by grafting 4T1 breast cancer cell line **(A-C)** Graph of flow-cytometry analyses in co-culture of 4T1 and RAW264.7 (n=3). (A) Expression of ABCG2 on cancer cell surface. (B) Expression of CD44. (C) Expression of EpCAM. **(D)** Graph of tumor volume change (eleven tumors in each treatment groups). 4T1 cells were implanted into the flank of BALB/c mouse by subcutaneous injection. 6mg/kg of CBP501 was administrated by i.v. injection (twice at first, eighth and fifteenth day, once at second, ninth and sixteenth day). 3mg/kg of CDDP was administrated by i.p. injection at second, ninth and sixteenth day. **(E)** Representative image of lungs from D. Arrows indicate metastatic foci. **(F)** Graphs of lung weight (left) and lung weight per body weight (right). Mice from D were measured bodyweight before dissection at twenty-fifth day. Then weights of lungs including metastatic foci were measured. **(G)** Overall survival curves in same treatment condition to D (final tumor volume 2000-4000mm^3^, 9 mice for CDDP, 11 mice for CDDP/CBP501, p=0.028 in Log-rank test). **(H)** Diagram summarizing effects of CBP501 in tumor microenvironment (cancer and macrophage interaction). An asterisk indicates statistical significance (TTEST, p<0.05). Two asterisks (TTEST, p<0.005). Three asterisks (TTEST, p<0.00005).N.S. = not significant (TTEST, p>0.05). Error bars indicate the standard deviation for A-C and F. Error bars indicate the standard error for D.

Many reports indicate that CSC-like cell formation at primary tumor sites promotes metastases [11, 12]. To evaluate the effects of CBP501 on metastases, a tumor xenograft model of the 4T1 mouse breast cancer cell line was employed. The cell line 4T1 is generally used as metastasis model because of its high metastatic potential [46]. First, 4T1 cells were added to the co-culture system with RAW264.7 cells. LPS stimulation increased ABCG2 expression in 4T1 cancer cells (Figure 7A). Additional CSCs markers, CD44 and EpCAM [47], were also increased by LPS stimulation in the co-culture system (Figure 7B, 7C). Expression levels of each of these three markers were suppressed by CBP501 treatment (Figure 7A-7C). These results indicate that formation of a CSC-like population by interactions between cancer cells and macrophages is a more universal event and that a 4T1 tumor graft is a suitable model to evaluate CBP501′s effect on metastases. Such an *in vivo* 4T1 tumor model showed no differences in tumor volume changes when comparing treatments with CDDP alone and CDDP/CBP501 combination (Figure 7D). However, the appearance of metastatic foci, associated with an increase in the weights of autopsied lungs, was suppressed by the CDDP/CBP501 combination treatment relative to CDDP alone (Figure 6E, 6F). Moreover, the overall survival curve was extended by the CDDP/CBP501 combination compared to CDDP alone (Figure 7G). These results suggest that suppression of a CSC-like population by CBP501 leads to an inhibition of metastases and extension of overall survival.

## DISCUSSION

Our results provide novel mechanism for the appearance of CSC-like properties in tumor cells in the tumor microenvironment. Activation of macrophages through their TLR2/4 leads to the release of cytokines such as IL-6 and TNF-α to adjacent cancer cells. These cytokines play a role in the appearance of “anchorage-independent cell proliferation” and “tumor initiation,” two specific CSC-like properties. In addition, juxtacrine interaction between cancer cells expressing VCAM-1 and macrophages expressing VLA-4 induces expression of the CSC-like cell marker ABCG2, which relates to drug resistance, on cancer cell surfaces. CaM inhibition by CBP501 could suppress these mechanisms of CSC-like cell formation by a mechanism that at least minimally includes inhibition of NFAT and NFκB nuclear translocation in macrophages (Figure 7H).

In this report, we focused mainly on; 1) the formation of CSC-like cells through both paracrine and juxtacrine interactions between cancer cells and macrophages; and 2) the suppressive effect of CBP501 on these mechanisms. However, paracrine interactions may drive various other conditions that also lead to tumor promotion. For example, tumor vascularization is one of the conditions promoted by TAMs. Expression of VEGF, Transforming growth factor-β (TGF-β) and other effectors by TAMs all promote tumor vascularization [11]. EMT, which is also related to CSC-like cell formation, is an important mechanism for promoting metastasis [12] and is also induced by TAMs through the production of various soluble factors [11, 48]. CBP501 treatment might broadly suppress not only CSC-like cell formation but also other TAMs-driven tumor progression events. Actually, when EMT induction was examined in a co-culture of the H1299 lung cancer cell line and a THP-1 derived macrophage cell line, it was possibly suppressed by CBP501 treatment (Supplementary Figure 7). Thus, various other mechanisms induced by cytokines could also be the targets of CBP501 treatment, which will be the focus of subsequent studies.

Our results indicate that CBP501 suppresses the ABCG2 expression on cancer cell surfaces that may be induced in a co-culture system upon stimulation with LPS or HMGB1. ABCG2 is the key drug resistance marker and one of the key markers of the CSC state [23, 24]. This expression of ABCG2 was induced by juxtacrine interactions between VCAM-1 on cancer cells and VLA-4 on macrophages. CBP501 reduced the expression levels of VCAM-1 on cancer cells and β1-integrin on macrophages. These membrane proteins are known to be targets of NFκB [38, 49]. Additionally, CBP501 had a suppressive effect on nuclear translocation of NFκB. Therefore, the reduction of VCAM-1 and β1-integrin by CBP501 treatment might result from suppression of NFκB nuclear translocation. One report indicates that calcium-binding CaM positively regulates the binding activity of integrin to its ligands [39]. In previous work, we showed that CBP501 binds to and inhibits CaM [13]. CBP501 might therefore suppress ABCG2 expression on cancer cells not only through reduction of expression levels of VCAM-1 and β1-integrin, but also through its suppression of integrin function. A recent report indicates that interactions between EphA4 on cancer cells and ephrin on macrophages leads to a CSC state for cancer cells without stimulation by external ligands such as LPS and HMGB1 [43]. This mechanism, and our discovery of ABCG2 induction, might have a synergistic role in CSC-like cell formation and maintenance.

TAMs are generally characterized as exhibiting features of two polar subtypes; M1 subtype is known to have anti-tumor activity whereas M2 has pro-tumor activity [1, 7]. However, it has been proposed to revise this simple categorization to include more subgroups especially in place of M2 type, namely M2a, M2b and M2c, given an increased variety of stimuli and phenotypes that have been observed for macrophages [44]. In our study, RAW264.7 cells were initially stimulated with IFN-γ and LPS, which were typical inducers of M1 type. However, as shown in Supplementary Figure 5 and Figure 2D-2H and 2K-M IFN-γ turned out not to be required in our system, therefore IFN-γ was removed from all the later experiments (Figure 3 and after) and HMGB1 was able to substitute for LPS. Furthermore, as shown in Figures 1C and 2M RAW264.7 cells expressed IL-10 at a high level in our model system. These facts are in accordance with the hypothesis that RAW264.7 in our system exhibits an M2b phenotype, for which IL-6, TNF and IL-10 are induced at high-levels by TLR stimulation, rather than an M1 phenotype for which IL-10 induction would be low and IL-6 and TNF would be highly expressed [45]. Tumor progression is often accompanied by appearance of a necrotic region that contains released HMGB1 [40]. Macrophages favor such condition [41]. In addition, CSCs are found to be abundantly localized in peri-necrotic areas, as is observed in human renal cell carcinoma [50]. Taken these together, these reports suggest that HMGB1-primed TAMs in the necrotic region of a tumor can function both as an anti-tumor M1-like macrophage and as a CSC-generating macrophage. Further studies are warranted to examine whether our findings that the CSC decrease caused by CBP501 in the system reported here are relevant in the tumor micro environment in humans.

In conclusion, we have shown that production of cytokines, such as IL-6, TNF-α and IL-10, was induced synergistically by combined CDDP/LPS stimulation of macrophages and that such induced cytokines are involved in CSC-like cell formation. These results indicate that although CDDP might have a cytotoxic effect for cancer cells, it might also induce CSC-like cell formation as a secondary effect through macrophage stimulation. In other words, treatment with CDDP might contribute to the selective growth of CSCs through its activation of TAMs. CDDP induced the production of IL-6 upon synergistic treatment with either LPS or IFN-γ. Although CDDP can activate macrophages by inducing MAPK signaling [51], how CDDP causes its synergistic effect on cytokine production, as is seen upon stimulation with either LPS or IFN-γ, remains unclear. Another important finding here is that cancer cells were able to prime the sensitivity of macrophages to combined CDDP/LPS stimulation. These points of interest need to be examined further in future studies. CBP501 mainly suppressed TLR2/4-mediated LPS- or HMGB1-stimulated signaling but not IFN-γ-stimulated signaling which may become important as IFN-γ signals are directed toward M1 type macrophges. Such activation of macrophages through TLR is implicated in the promotion of metastasis [52–54]. In fact, CBP501 suppressed metastasis in a tumor xenograft model of the 4T1 mouse breast cancer cell line when used in combination with CDDP. This result suggests the possibility that the combined treatment with CDDP/CBP501 might have efficacy against breast cancer. Future studies in other preclinical models, such as the mouse mammary tumor virus (MMTV)-induced spontaneous mammary tumor model, will be needed to elucidate this possibility. Taken together, these results suggest that combined treatment with CDDP/CBP501 might possibly offer new, unanticipated advantages in anti-tumor therapy, at least for breast cancer, through a mechanism that entails the suppression of interactions between TAMs and cancer cells with concomitant suppression of sequential CSC-like cell formation in the tumor microenvironment.

## MATERIALS AND METHODS

### Cell culture and reagents

Mouse cancer cell lines, a mouse macrophage cell line, a human NSCLC cell line and a human monocyte cell line were cultured in following media, each supplemented with 10% fetal bovine serum (Life Technologies, San Diego, CA) and 1% Penicillin-Streptomycin (GIBCO, Pittsburgh, PA) at 37°C with 5% CO_2_/air. The media used was RPMI1640 (Sigma-Aldrich, St. Louis, MO) for Ex3ll, 4T1, NCI-H1299 and THP-1; DMEM (Sigma-Aldrich) for RAW264.7. CDDP, LPS, W7, Calmidazolium and Phorbol 12-myristate 13-acetate (PMA) were purchased from Sigma-Aldrich. IFN-γ was purchased from Peprotech (Rocky Hill, NJ). HMGB1 was purchased from Iwai chemical (Tsukuba, Japan). HMR1031 was purchased from Funakoshi (Tokyo, Japan). Transwell permeable supports were purchased from Corning (Lowell, MA). Ex3ll was purchased from JCRB (Osaka, Japan), who had performed short tandem repeat (STR) analysis to authenticate the identity of this cell line. 4T1, NCI-H1299, RAW264.7 and THP-1 cell lines were purchased from ATCC, who had performed STR analysis to authenticate the identity of each of these cell lines. All cell lines were used within three months after thawing.

### Antibodies

Antibodies were purchased from the following sources: anti-phospho-NFATC2, anti-NFATC1, anti-NFATC4, anti-TLR2, anti-TLR4, anti-a4integrin, anti-b1integrin (SantaCruz, Dallas, TX); anti-IQGAP1, anti-b-actin, anti-NFkB, anti-HSP90, anti-HH3, anti-phospho-IKKa/b, anti-IkB, anti-Vimentin, anti-E-cadherin, anti-Snail (Cell Signaling Technology, Beverly, MA); anti-TNFa, anti-VCAM-1, anti-EpCAM, anti-CD44, anti-ABCG2, anti-CD11b (Abcam, Cambridge, MA), anti-CD45 and anti-CD34 (BectonDickinson: BD, Frankrin Lake, NJ).

### Lentivirus infection

IL-6, VCAM-1, b1integrin and control shRNA lentivirus particles were purchased from SantaCruz. Lentivirus infection was performed according to manufacturer's instructions. Cells (50% confluence) were treated with 5mg/ml Polybrene (SantaCruz) prior to virus infection. One day after addition of virus, the cells were transferred to fresh medium and cells were selected by treatment with Puromycin (SantaCruz).

### Cell fractionation

Cell fractionations were prepared using a commercially available kit (ATTO, Tokyo, Japan) according to the manufacturer's instructions.

### Western blot analysis

The cells were harvested and lysed (30 min on ice) in lysis buffer [50 mM Tris-HCl (pH 8.0), 5 mM EDTA (pH 8.0), 100 mM NaCl, 0.5% NP-40, 2 mM DTT, 50 mM NaF, 1 mM Na_3_VO_4_, 1 mM microcystin, proteinase inhibitors cocktail (Roche, Mannheim, Germany)]. The lysates were clarified by centrifugation (20600g, 4°C), and the supernatants were assayed for protein content using the detergent-compatible protein assay kit (Bio-Rad, Hercules, CA) according to the manufacturer's instructions. The whole cell lysates (30-60 mg) were run on 7.5-12% SDS-PAGE. Protein from each gel was transferred onto a polyvinylidene difluoride, (PVDF), membrane (Bio-Rad). The membrane was blocked at room temperature for 1 h in TBST (10 mM Tris-HCl [pH 8.0], 150 mM NaCl and 0.05% Tween-20) containing 1 % Block Ace (DS pharma) and was incubated with primary antibody overnight at 4°C. The membrane was incubated further with anti-peroxidase conjugated secondary antibody (Cell Signaling) for 1 h at room temperature after washing, and the signals were detected using the enhanced chemiluminescence detection system (ECL Advance Western Blotting Detection Kit, GE Healthcare). Detected bands were quantified using Lumino-meter LAS-4000 instrument (Fujifilm, Tokyo, Japan).

### Cell cycle analysis

Cells were plated in 24-well plates and incubated for 24 h. The cells were treated with or without CDDP and with or without (+/−) CBP501 at the indicated concentrations for the indicated times. The cells were harvested and stained with Krishan's solution (0.1% sodium citrate, 50 mg/ml propidium iodide, 20 mg/ml RNase A, 0.5% NP-40). Stained cells were analyzed by FACSCalibur (BD) using CELLQuest software (BD).

### Cell viability assay

RAW264.7 cell lines that had been cultured to log phase were harvested and plated on a 96-well microplate at a density of 10000 per well. Cells were incubated with drugs for 48 h. After incubation, cell viability assays were carried out using a Cell Counting Kit-8 (Dojindo).

### ELISA assay

120000 cells from a mouse cancer cell line and 100000 cells of RAW264.7 were seeded into same well of 48 well plate (FALCON, Pittsburgh, PA). Alternatively, 100000 cells of NCI-H1299 were added to 100000 cells of THP-1 that had been stimulated with PMA (30 ng/ml) for 72 hours. After 24 hours of co-incubation, cells were transferred into fresh media and the indicated ligands or drugs were added. After an additional 21-24 h, cell culture supernatants were collected for ELISA assays. All ELISA assays were performed using commercially available kits (R and D system, Minneapolis, MN).

### LDV-FITC binding assay

Raw264.7 cells were treated with LPS and CBP501 at indicated concentrations for 24 hours. Raw264.7 cells (1 × 10^6^ cells in 50 μl), which were suspended in HBSS (with 1 mM Ca^2+^Mg^2+^) that had been supplemented with 20 mM HEPES and 0.5% FBS, were incubated with LDV-FITC (Funakoshi, 3 nM) for 30 min at 37°C. The amount of LDV-FITC binding to cells was measured by Cytoflex (BeckmanCoulter, Brea, CA).

### Flow-cytometric analysis

Cells were cultured in indicated conditions (2 hr pretreatment of CBP501 was performed for CBP501 treatment groups.). Cells were incubated for 30 minutes in 100 μl of fluorophore-conjugated anti-mouse antibodies. Data acquisition was performed on Cytoflex, using FlowJo software version 10 (Tree Star) for the analysis.

### Spheroid formation assay

Cells were seeded into 24-well ultra low-attachment surface plates (Corning) at a density of 1500 cells/well and were cultured in serum-free RPMI1640 supplemented with 1.5% B27 (Life technologies), 20 ng/ml mouse recombinant EGF and 10 ng/ml FGF (Peprotech). Conditioned medium was prepared identically, but without adding EGF and FGF. Spheroids formed within 5 to 6 days after seeding.

### Mouse tumor graft model

C57BL/6, BALB/c and SCID mouse lines (age 5 weeks) were purchased from Charles River (Yokohama, Japan). Ex3ll cancer cells (total count 8×10^6^ cells) were transplanted subcutaneously into the flanks of C57BL/6 mice. Drug administration (7.5 mg/kg of CBP501 i.v. injection, 3 mg/kg of CDDP i.p. injection) was started when grafted tumors reached an average volume of around 400 mm^3^. 4T1 cancer cells (total count 5×10^5^ cells) were transplanted subcutaneously into the flanks of BALB/c mice. Drug administration (6 mg/kg of CBP501 i.v. injection, 3 mg/kg of CDDP i.p. injection) was started when grafted tumors reached an average volume of around 100 mm^3^. The volumes were calculated using the following formula: volume (mm^3^) = [width^2^ (mm) × length (mm)] / 2. The relative tumor volume was expressed as theV_t_/V_0_ index, where V_t_ is the tumor volume on a given day and V_0_ is the volume of the same tumor just before the first treatment (i.e., initial tumor volume). Animals were housed in accordance with guidelines from the Association for the Assessment and Accreditation of Laboratory Animal Care International, and the protocols were approved by the institutional animal care committee of CanBas Co. Ltd.

### Isolation of mouse peritoneal macrophages

C57BL6 mice (Charles River) were sacrificed and 7 ml of ice cold PBS was injected into the intraperitoneal cavity. The abdomen was gently massaged for 3 min and the injected PBS, now containing macrophages, was recovered by aspiration. Upon centrifugation, the collected cell pellets were washed twice with DMEM containing 10% FBS. The collected peritoneal macrophages were then plated into dishes or multiwell plates and cultured at 37°C in a humidified incubator supplemented with 5% CO_2_.

### Statistical analysis

Data analysis was performed using Microsoft Excel. Bar graphs represent means ± SEM or ± SD, as indicated. Student *t* test or Log-rank test was performed, as indicated, to assess statistical significance.

## SUPPLEMENTARY MATERIALS FIGURES


