# Supplementary material for: CBP501 suppresses macrophage induced cancer stem cell like features and metastases
Source: Oncotarget. 2017 Jul 17;8(38):64015–31. doi: 10.18632/oncotarget.19292 (PMC5609981; doi:10.18632/oncotarget.19292)
Supplement: Supplementary file 1 [file oncotarget-08-64015-s001.pdf]

CBP501 suppresses macrophage induced cancer stem cell like features and metastases

SUPPLEMENTARY MATERIALS

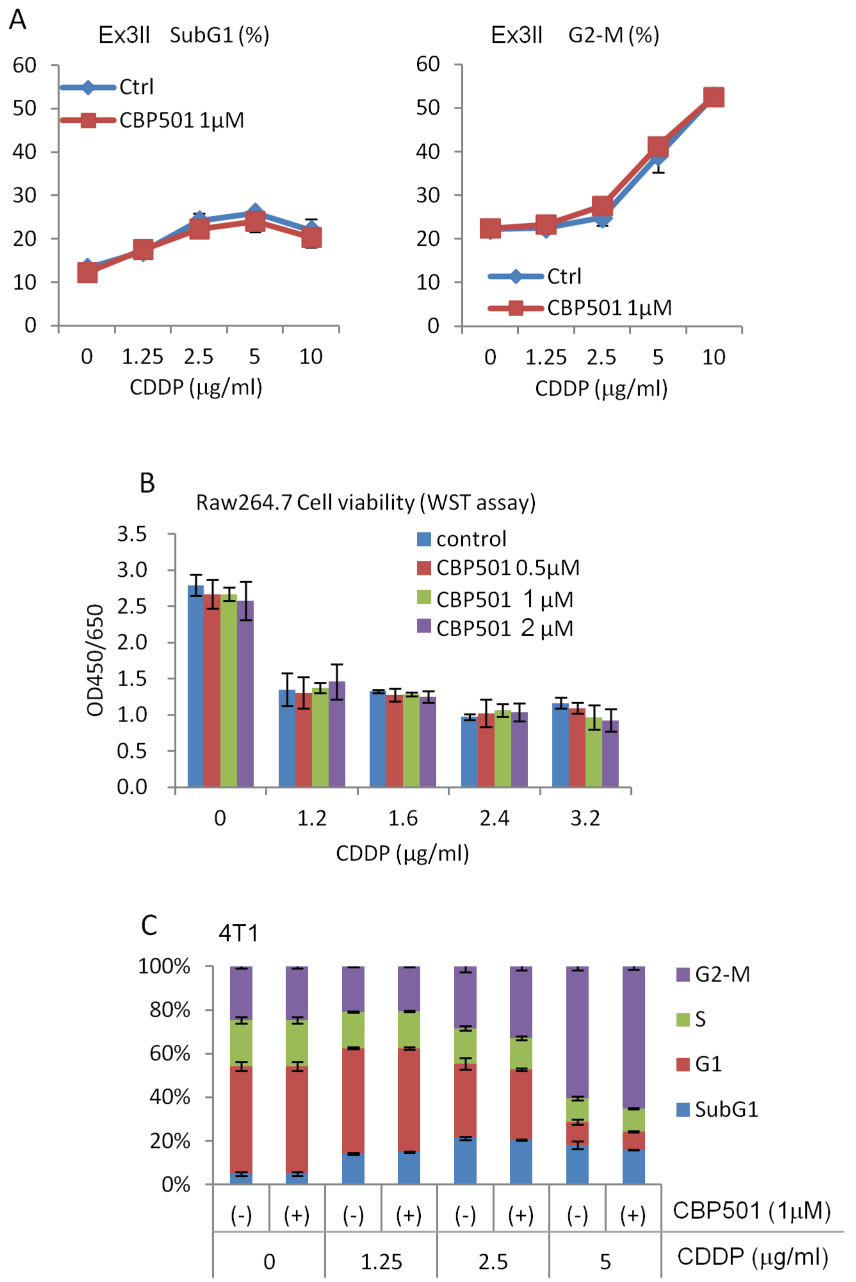

Supplementary Figure 1: CBP501 does not increase CDDP induced cyto-toxicity in Ex3ll, RAW264.7 and 4T1 cell lines. **(A)** Flowcytometry analyses for cell cycle distributions for subG1 (left graph) and G2-M phase (right graph) (n=3). 1 h treatment of drugs. Medium was replaced and cultured 48 hours. **(B)** WST assay (n=3). 48 h treatment of drugs. **(C)** Flowcytometry analyses for cell cycle distributions. Error bars indicate the standard deviation.

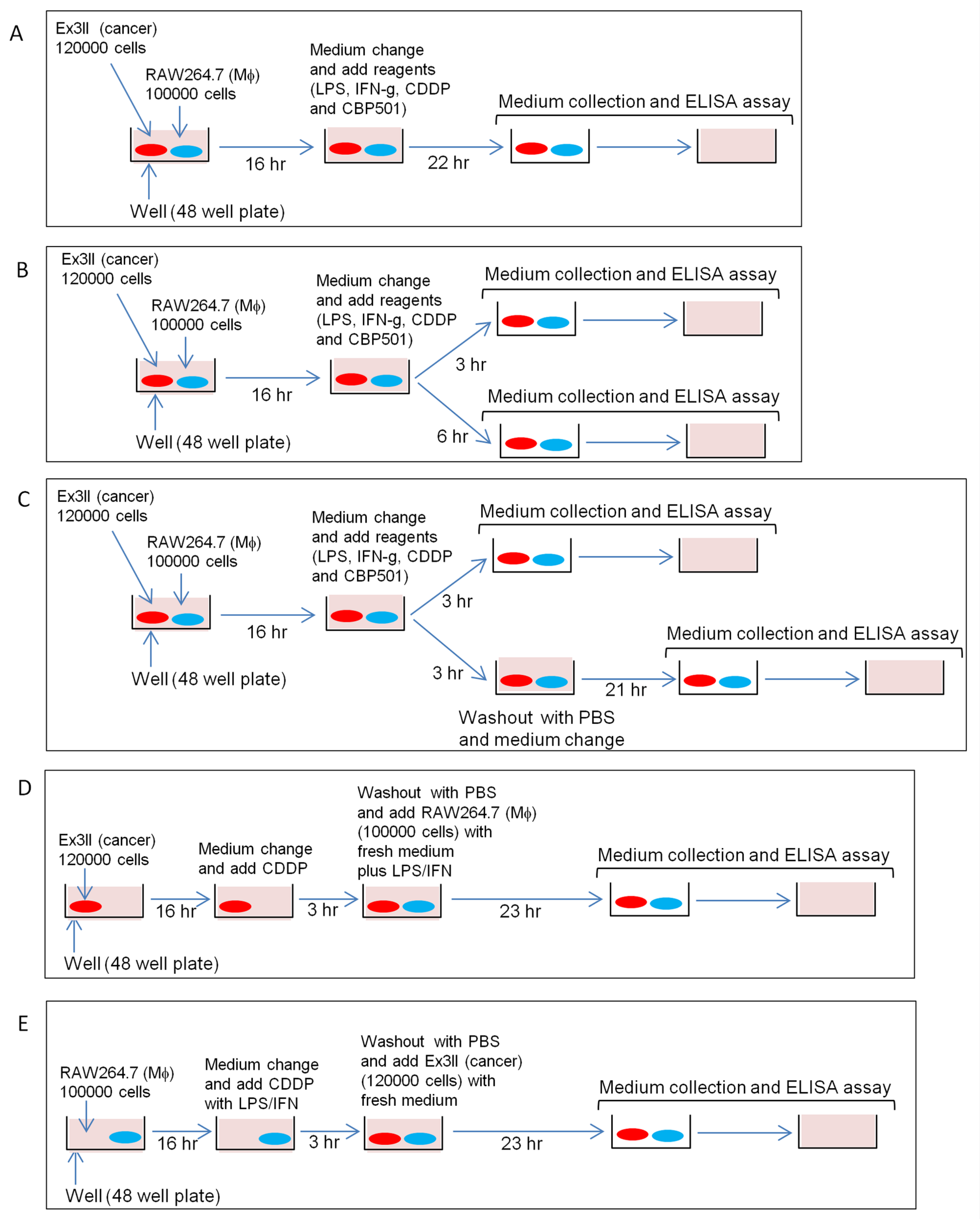

Supplementary Figure 2: Summary of co-culture assay scheme. **(A)** Co-culture assay scheme for Figure1 A-C. **(B)** Co-culture assay scheme for Figure1 D. **(C)** Co-culture assay scheme for Figure1 E. **(D)** Co-culture assay scheme for Figure2 A. **(E)** Co-culture assay scheme for Figure2 C.

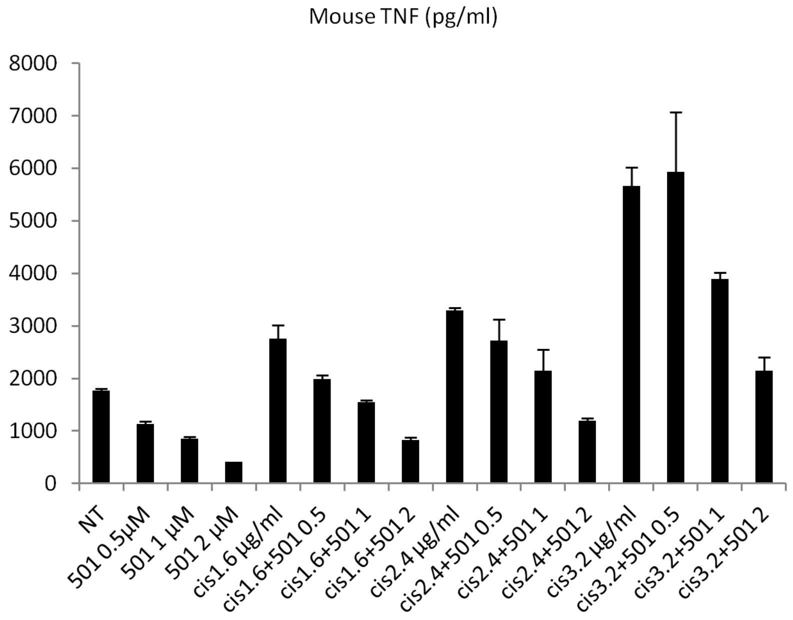

Supplementary Figure 3: CBP501 suppresses CDDP induced TNF-α production in co-culture system of Ex3ll and RAW264.7 cell lines. 48 h culture supplemented with 0.39 ng/ml of IFN-g and 0.078 ng/ml of LPS (n=3). Error bars indicate the standard deviation. cis:CDDP

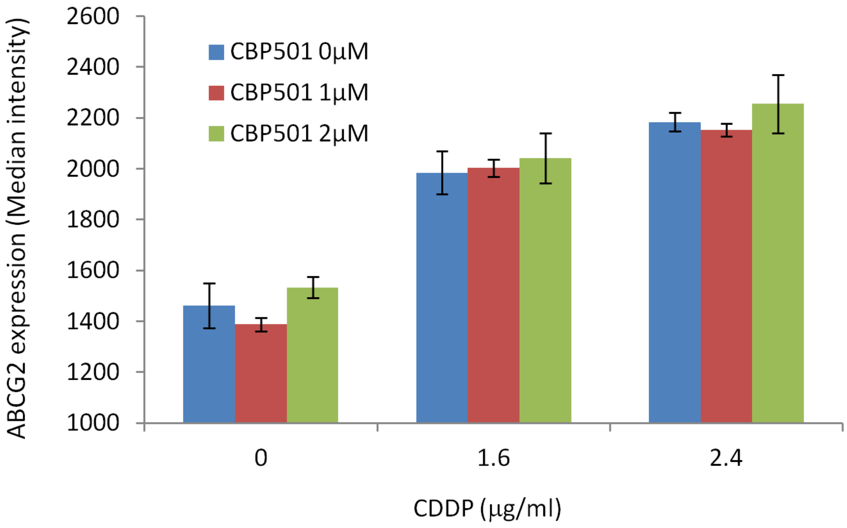

Supplementary Figure 4: CBP501 does not suppress CDDP induced ABCG2 expression in single culture of Ex3ll. Flowcytometry analyses for ABCG2. 48 hours culture supplemented with 0.39 ng/ml of IFN-g and 0.078 ng/ml of LPS (n=2). Error bars indicate the standard deviation.

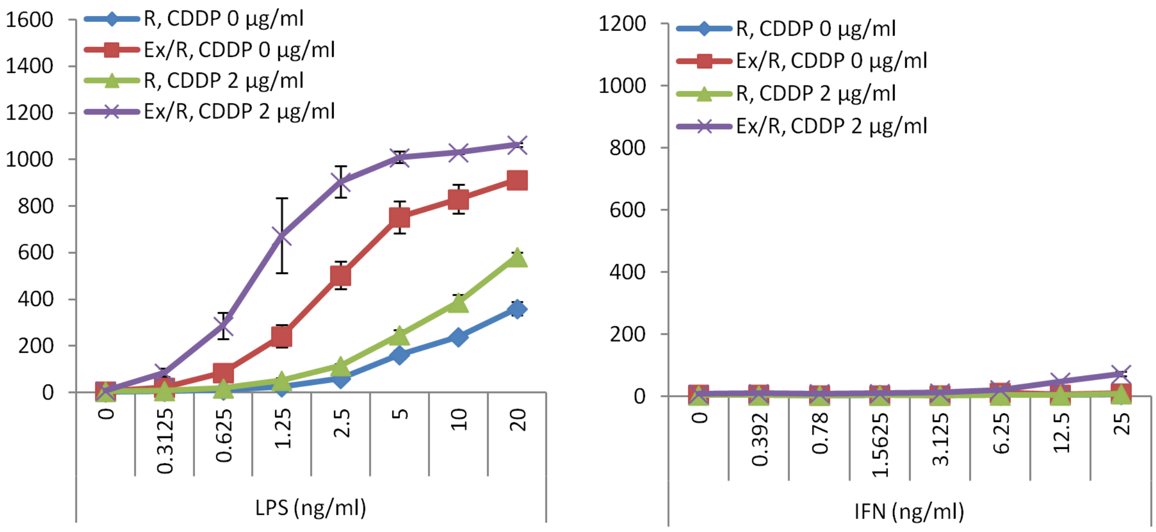

Supplementary Figure 5: LPS induced robust production of IL-6 but IFN-γ did not induce IL-6 production in co-culture system. Both left and right graphs show IL-6 production (n=3). R means single culture of RAW264.7 (100000 cells). Ex/R means co-culture of Ex3ll (120000 cells) and RAW264.7 (100000 cells). Error bars indicate the standard deviation.

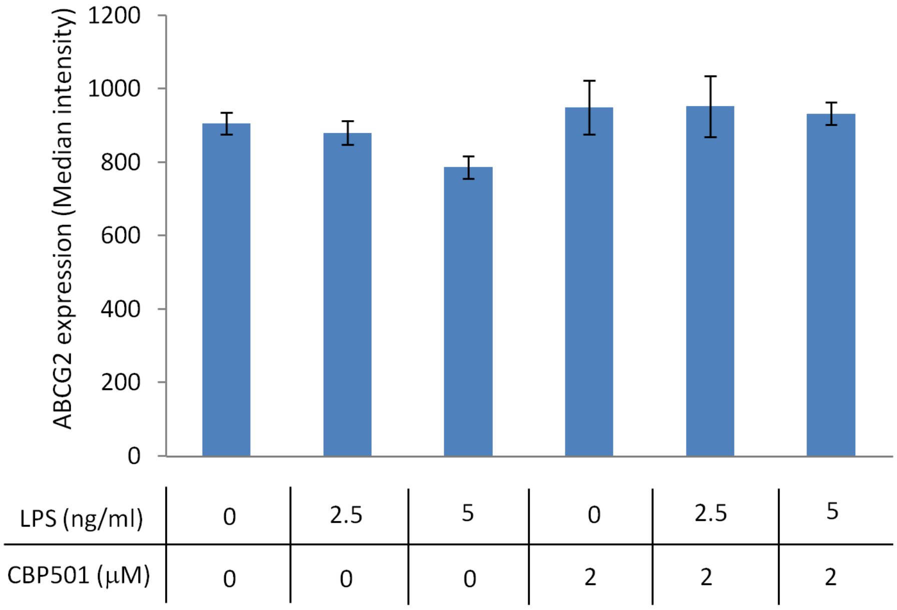

Supplementary Figure 6: Conditioned medium from RAW264.7 did not increase ABCG2 expression in Ex3ll. Flowcytometry analyses for ABCG2. Conditioned medium from LPS stimulated RAW264.7 (24 h) was added to Ex3ll and cultured 48 hours (n=3). Error bars indicate the standard deviation.

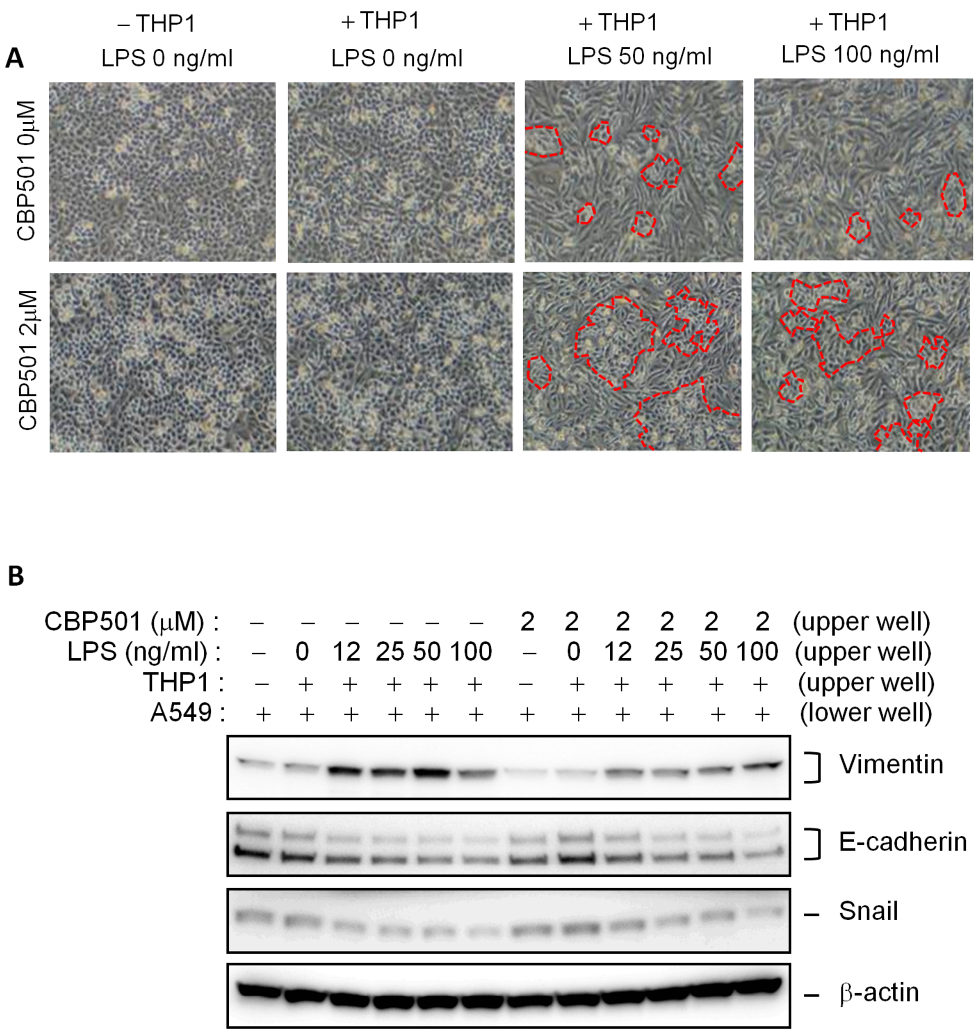

Supplementary Figure 7: EMT induction in co-culture of H1299 lung cancer cell line and THP-1 derived macrophage cell line was suppressed by CBP501 treatment. (A, B) Trans-well experiments (48 hours culture). THP-1 is in upper-well. H1299 is in lower-well. (A) Microscopic images of lower-well (H1299). Red broken line is surrounding grape-shape cells (epithelial feature) in LPS stimulating condition. CBP501 treatment suppressed formation of spindle shape (mesenchymal feature). (B) Western bolt analyses for EMT markers.
